# Reirradiation + hyperthermia for recurrent breast cancer en cuirasse

**DOI:** 10.1007/s00066-017-1241-7

**Published:** 2017-12-20

**Authors:** Sabine Oldenborg, Coen R. N. Rasch, Rob van Os, Yoka H. Kusumanto, Bing S. Oei, Jack L. Venselaar, Martijn W. Heymans, Paul J. Zum Vörde Sive Vörding, Hans Crezee, Geertjan van Tienhoven

**Affiliations:** 1 Department of Radiation Oncology, Z1-215, Academic Medical Center, University of Amsterdam (AMC), Meibergdreef 9, P.O. Box 22660, Amsterdam, The Netherlands; 2 Department of Radiation Oncology, Institute Verbeeten (BVI), Tilburg, The Netherlands; 3 Department of Epidemiology and Biostatistics, VU University Medical Center, Amsterdam, The Netherlands

**Keywords:** Treatment outcome, Hyperthermia, induced, Palliation, Radiation-sensitizing agents, Drug-related side effects and adverse reactions, Behandlungserfolg, Induzierte Hyperthermie, Palliation, Strahlensensibilatoren, Medikamentenabhängige Nebenwirkungen und unerwünschte Reaktionen

## Abstract

**Background and purpose:**

Patients with irresectable locoregional recurrent breast cancer en cuirasse (BCEC) do not have effective curative treatment options. Hyperthermia, the elevation of tumor temperature to 40–45 °C, is a well-established radio- and chemotherapy sensitizer. A total of 196 patients were treated with reirradiation and hyperthermia (reRT+HT) at two Dutch institutes from 1982–2005. The palliative effect was evaluated in terms of clinical outcome and toxicity.

**Patients and methods:**

All patients received previous irradiation to a median dose of 50 Gy. In all, 75% of patients received 1–6 treatment modalities for previous tumor recurrences. ReRT consisted of 8 × 4 Gy given twice a week or 12 × 3 Gy given four times a week. Superficial hyperthermia was added once or twice a week. Tumor area comprised ≥½ of the ipsilateral chest wall.

**Results:**

Overall clinical response rate was 72% (complete response [CR] 30%, partial response [PR] 42%, stable disease [SD] 22%, progressive disease [PD] 6%). The local progression-free rate at 1 year was 24%. Median survival was 6.9 months. Forty-three percent of our patients with CR, PR, SD after treatment remained infield progression-free until death or last follow-up. Acute ≥grade 3 toxicity occurred in 33% of patients, while late ≥grade 3 toxicity was recorded in 14% of patients. Tumor ulceration prior to treatment had a negative impact on both clinical outcome and toxicity.

**Conclusion:**

ReRT+HT provides sustainable palliative tumor control, despite refractory, extensive tumor growth. Compared to currently available systemic treatment options, reRT+HT is more effective with less toxicity.

**Electronic supplementary material:**

The online version of this article (10.1007/s00066-017-1241-7) contains supplementary material, which is available to authorized users.

## Zusammenfassung

**Hintergrund und Fragestellung:**

Für Patienten mit inoperablen lokoregionalen Rückfällen von Brustkrebs in Form eines Cancer en cuirasse (BCEC) gibt es keine effektiven kurativen Behandlungsoptionen. Die Hyperthermie, bei der die Tumortemperatur auf 40–45 °C erhöht wird, ist eine etablierte Methode zur Radio- und Chemotherapiesensibilisierung. Insgesamt 161 Patientinnen wurden in zwei niederländischen Kliniken von 1982–2005 mit Rebestrahlung und Hyperthermie (reRT+HT) behandelt. Der palliative Effekt wurde anhand von klinischem Verlauf und Toxizität bewertet.

**Patienten und Methoden:**

Alle Patienten hatten eine vorangegangene Bestrahlung mit einer medianen Dosis von 50 Gy erhalten. Insgesamt wurden 75 % der Patienten mit 1–6 Behandlungsmaßnahmen wegen vorhergehender Rückfälle behandelt. Die reRT erfolgte mit 8 × 4 Gy 2‑mal pro Woche oder 12 × 3 Gy 4‑mal pro Woche. Die Oberflächenhyperthermie wurde 1‑ bis 2‑mal pro Woche durchgeführt. Die Tumorareale umfassten ≥½ der ipsilateralen Brustwand.

**Ergebnisse:**

Die klinische Ansprechrate lag insgesamt bei 72 % (vollständiges Ansprechen [CR] 30 %, partielles Ansprechen [PR] 42 %, stabile Erkrankung [SD] 22 %, progressive Erkrankung [PD] 6 %). Die lokale progressionsfreie Rate betrug nach einem Jahr 24 %. Das mediane Überleben lag bei 6,9 Monaten. Nach Behandlungsende zeigten bis zum Tod oder bei der letzten Nachbeobachtung 43 % unserer Patienten mit CR, PR, SD keine Progression im Bereich des Behandlungsfelds. Akute ≥Grad-3-Toxizitäten traten bei 33 % auf, späte ≥Grad-3-Toxizitäten bei 14 % der Patienten. Eine Tumorulzeration vor Behandlungsbeginn hatte einen negativen Einfluss auf den klinischen Verlauf sowie auf die Toxizität.

**Zusammenfassung:**

Eine reRT+HT ermöglicht bei BCEC eine anhaltende palliative Tumorkontrolle trotz refraktärem ausgedehntem Tumorwachstum. Im Vergleich zu aktuell verfügbaren systemischen Behandlungsoptionen ist die reRT+HT effektiver und weniger toxisch.

Carcinoma en cuirasse (also known as scirrhous carcinoma or pachydermia) presents a form of breast cancer which involves extensive areas of the (sub)cutaneous chest wall. It frequently crosses the midline and spreads to the dorsal, abdominal, or groin regions. Ulceration is often present [1]. As these type of tumor cells lie embedded in a matrix of extensive fibrosis and poor vascularity, chemotherapeutic agents cannot reach them in effective concentrations [2]. An additional treatment challenge is presented by patients with recurrent breast cancer en cuirasse (BCEC) in previously irradiated area, as the dysfunctional microvasculature caused by previous radiation and/or surgery adds to the tumor’s resistance to both radiation therapy (RT) and chemotherapy [3, 4]. In addition, the reirradiation (reRT) dose that can be given without a risk of unacceptable toxicity is lower than considered adequate [5–7]. Reports on treatments options for patients with cancer en cuirasse are lacking.

Effects of systemic treatment modalities on locoregional disease are rarely described. Three phase II studies reported overall response rates (ORR = complete response [CR] + partial response [PR]) for locoregional disease separately, although in a very small numbers of patients. A trial of capecitabine and paclitaxel resulted in a clinical benefit rate (ORR including stable disease [SD] ≥6 months) of 62% (16/26) for lymph nodes and 67% (4/6) for skin metastases [8]. A trial using albumin-bound paclitaxel showed an ORR of 30% (3/10) for skin metastases and 20% (7/35) for affected lymph nodes [9]. The third trial on vinorelbine and cisplatin reported ORR rates of 59% (10/17), 46% (6/13), and 44% (4/9) for metastases in skin/chest wall, lymph nodes, and breast, respectively. CR rates were 24%, 23%, and 11%, respectively [10].

The literature on toxicity of systemic therapy for locoregional recurrent breast cancer is much more common; 19 articles (35 studies) on phase II–III studies were published between 2007 and 2015 on refractory inoperable locoregional recurrent/metastatic breast cancer. Seven different systemic therapy regimens were evaluated and included toxicity analyses, but not locoregional response rates. Specific and overall grade 3 + 4 toxicity rates are reported in Supplement 1 and 2, including references. Overall grade 3 + 4 toxicity rates varied from 27–89%. Up to 33% treatment-related deaths occurred, 0–35% of patients had to discontinue treatment because of toxicity and another 1–84% could not complete treatment as planned because of toxicity and required dose omissions, reductions, or modifications.

Hyperthermia (HT), the elevation of tumor temperature to 40–45 ˚C, is a well-established radiation and chemotherapy sensitizer. It is known to inhibit DNA repair processes, affect tumor blood flow and oxygenation, and cause direct cytotoxicity to cells that are acidotic and nutrient deprived [11–18]. The combined results of five phase III trials demonstrated a significant 26% increase of complete response rates and a 20% improvement of the 3‑year local control (LC) rate when hyperthermia was added to reirradiation for patients with locoregional recurrent breast cancer in previously irradiated areas [4]. A meta-analysis by Datta et al. [19] confirmed these results. CR rate was improved from 38% for RT alone to 60% for RT+HT, and 66% after reRT+HT.

Our study only includes patients with BCEC in a previously irradiated area, resistant to previous treatments. Our aim is to evaluate the palliative effect of reRT+HT for this patient population in terms of tumor remission and incidence of ≥grade 3 side effects.

## Patients and methods

### Patients

In accordance with the Dutch National Guideline for Breast Cancer, patients with irresectable locoregional recurrent breast cancer in a previously irradiated area are treated with reRT+HT [20]. Currently, the Academic Medical Center of Amsterdam (AMC) and the Institute Verbeeten (BVI) treat approximately 70 new patients with recurrent breast cancer each year.

For the current study, patients with BCEC were included from 1982 up to 2006 to enable long-term follow-up (FU). BCEC patients were identified according one of the following criteria: (1) diffuse (sub)cutaneous tumor growth ≥¾ ipsilateral chest wall ± extension to back, abdomen, axilla, supraclavicular area and/or contralateral side, or (2) >½ but <¾ ipsilateral chest wall + extensive growth beyond this area. A total of 169 patients with BCEC (155 from AMC and 14 from BVI) were identified from our databases. The current study reports on the retrospective analyses of those 169 patients.

Data were collected from the radiation therapy and hyperthermia patient charts. In case of missing follow-up data, questionnaires were sent to referring specialists, general practitioners, and/or the relevant district or counsel register.

All patients received previous radiation, overlapping with the current reRT field. Ninety-four percent of the patients had also received one or more lines of systemic therapy in the past, either as primary adjuvant treatment, or as treatment for previous recurrent disease, distant metastases, or both. Seventy-five percent of the patients were treated for one or more previous locoregional recurrences with surgery, radiation, systemic therapy, or a combination of treatment modalities before the start of reRT+HT (Table 1).

**Table 1:** Previous treatments

	Percentage (*N*)	Median of Gy (range of Gy)
**Primary local treatment**		
*Surgery* ^*a*^	84% (139)	
BCT	35% (58)	
Mastectomy	44% (73)	
Other	5% (8)	
*Radiation*	82% (139)	
Total dose (excl. boost)		50 (20–62.5)
Additional boost^b^	69% (86)	
Total dose boost^c^		15 (4–44.7)
**Treatment for locoregional recurrent disease**		
*Systemic treatment*	68% (115)	
Chemotherapy	17% (28)	
Hormone therapy	21% (35)	
Both	31% (52)	
*Surgery*	27% (45)	(1–3 episodes)
Salvage mastectomy	14% (24)	
Chest wall resection	5% (9)	
Local excision	19% (32)	
Other	10% (17)	
*Radiation*	20% (33)	
Total dose (excl. boost)^d^		50 (30–62.5)
Fraction dose^e^		2 (2–8)
Additional boost^f^	56% (18)	
Total dose boost^g^		16.8 (10–27)

*BCT* breast conserving therapy, *excl.* exclusive, *N* number

^a^Missing for 1 patient

^b^Missing for 16 patients

^c^Missing for 23 patients

^d^Missing for 1 patient

^e^Missing for 15 patients

^f^Missing for 3 patients

^g^Missing for 1 patient

The entire area containing locoregional tumor was considered as the target volume for the end-point analysis. Characteristics of the current disease episode are summarized in Table 2.

**Table 2:** Patient and treatment characteristics at time of reRT+HT for recurrent BCEC

Characteristics	Percentage (*N*)	Median (range)
*Median FU time*		7 (0.1–67) months
*Median age at current treatment*		58 (28–87) years
*Median TI primary tumor—reBCEC*		43 (4–463) months
*Median TI primary RT—reRT*		35 (2–464) months
*Presence/history of DM*	45% (76)	
*Presence/history of regional disease*	49% (83)	
*Presence/history of contralateral disease*	66% (112)	
*Previous LR (1–6 episodes per patient)*	75% (127)	
*Tumor area current reBCEC*		
1) ≥¾ chest wall	46% (78)	
2) >½ but <¾ chest wall	54% (91)	
*Lymphangitis*	67% (113)	
*Ulceration* ^*a*^	52% (87)	
*ReRT dose*		
12 × 3 Gy	7% (12)	
8 × 4 Gy	6%1 (103)	
8–10 × 4 Gy	11% (18)	
Other (16; 20 × 2/6 × 2.5/5–8 × 3/1–7 × 4 Gy)	21% (36)	
*ReRT technique*		
Stanford^b^	10% (16)	
González^c^	34% (57)	
Multiple electron fields	13% (22)	
Locoregional	36% (60)	
Local	8% (14)	
*Systemic treatment* ^*d*^	59% (99)	
Chemotherapy	37% (63)	
Hormone therapy^e^	32% (54)	
*Tumor present outside current RT field* ^*f*^	22% (25)	

*N* number,* FU* follow-up, *TI* time interval, *BCEC* breast cancer en cuirasse, *reBCEC* current episode of recurrent breast cancer en cuirasse, *DM* distant metastases, *LR* locoregional recurrent disease, *RT* radiation therapy, *reRT* reirradiation, *HT* hyperthermia

^a^Missing for 1 patient

^b^Irradiate the whole chest wall with anterior–posterior/posterior–anterior photon fields for the lateral chest wall, and abutted anterior–posterior electron fields for the anterior chest wall

^c^Irradiation using lateral opposing photon fields to cover the anterior and/or posterior chests wall, and abutted lateral electron fields to cover the lateral chest wall

^d^In addition to the reRT+HT, given before, during or after the reRT+HT period, but indicated and given for the same disease episode

^e^Missing for 1 patient

^f^Missing for 56 patients

### Treatment

#### Radiation therapy

At AMC, patients were irradiated using a standard schedule of 8 fractions of 4 Gy given twice a week to a total dose of 32 Gy [4, 21]. At BVI, the standard reRT schedule consisted of 12 fractions of 3 Gy given four times a week to a total dose of 36 Gy (Table 2). Treatment fields were individualized for each patient. A minimum surface margin of 3–5 cm around the visible tumor was applied. Most patients (57%) received whole chest wall radiation. Other patients were treated with abutted anterior posterior-posterior anterior photon and/or anterior posterior electron fields. If regional lymph nodes were affected, these were also included in the target area. Typically the upper border of the radiation field was at the level of the coracoid process, or included the periclavicular area in case of regional recurrent disease. A bolus was applied to reach the most superficial layers of the skin. Thickness was determined by radiation technique and energy and adjusted according to tumor depth for each patient individually. Parts of the tumor areas that were not previously irradiated received conventional high dose RT without HT.

#### Hyperthermia

HT was given once a week at AMC and twice a week at BVI, starting within 1 h after radiation therapy. Heat was induced electromagnetically, using externally applied contact flexible microstrip applicators (CFMA), operating at 434 MHz [22]. Six patients were treated with a 70 MHz CFMA [23]. Treatment fields covered the entire target area. For very large tumor areas, the number of HT sessions were split to two weekly sessions at AMC and four at BVI. This enabled the use of multiple HT fields to cover the entire target volume. Aim temperature was 41–43 °C for one hour. For all patients, temperatures were measured with multisensory thermocouple probes on the skin and, if feasible or preferable, invasively using a thin flexible subcutaneous catheter.

### Endpoints and data analysis

#### Treatment response

Treatment response was assessed clinically, using the RECIST (response evaluation criteria in solid tumors) criteria [24]. The maximum clinical response at any time after reRT+HT was reported. In case of patients with multiple tumor locations, the location with the worst response rate was recorded and used for further analyses.

Eight patients had missing data on the status of macroscopic disease after treatment and were not included in the response analysis, but were included in the survival and toxicity analyses.

#### Local (infield) progression-free interval

Both the local (infield) progression-free interval (LPFI) and overall survival (OS) were calculated from the date of the first reRT fraction. Duration of LPFI and survival were analyzed by the actuarial method of Kaplan and Meier [25]. Local progression was defined as infield progression after CR, PR, or SD. PD was considered an event for LPFI at the zero timepoint. Patients dying without local progression, or alive without local progression at last FU, were censored at the date of death or last FU, respectively. Last FU was the last date with information on locoregional disease status. Fourteen patients did not have follow-up data on locoregional disease status and were not included in the LPFI analysis, but were included in the survival and toxicity analyses. For OS, patients known to be alive at last FU were censored at that date.

#### Toxicity

Grade 3–5 acute and late toxicity were assessed according to The National Cancer Institute’s Common Terminology Criteria for Adverse Events, (CTC-AE) version 3.0 [26]. To avoid bias, aggravation of pre-existing toxicity as well as toxicity of uncertain cause were considered to be related to the present treatment and scored accordingly. Toxicity was considered acute when occurring within 3 months after the start of reRT+HT and late when occurring >3 months after the start of reRT+HT. Late toxicity was calculated by the actuarial method of Kaplan and Meier [25] from the start of reRT+HT to the date of first ≥grade 3 toxicity notification. Patients without late toxicity were censored at date of last FU. Four patients did not have data on acute and late toxicity and were excluded from toxicity analysis but were included in all other analyses.

#### Statistics

Statistical analysis was carried out using the statistical program R version 2.13.0 and SPSS version 23 (SPSS Inc., Chicago, IL, USA). A multivariable analysis was done for overall response rates (ORR; using binary logistic regression), LPFI (Table 3), and ≥grade 3 toxicity (Cox regression). All multivariable tests were carried out in backward Wald stepwise manner [27]. Only variables available for at least 80% of the population were tested. The 2‑tailed Pearson correlation test was used to determine correlation coefficients. Variables with strong (>70%) correlations were not entered in the same multivariable model. The continuous variables were checked for linearity by using spline regression curves and spline coefficients tested for non-linearity. Variables included in the models were the following: time interval to recurrence, age, presence/history of distant metastases (DM), presence/history regional disease, presence/history of contralateral disease, current episode of recurrent breast cancer en cuirasse (reBCEC) ≤¾ : >¾ chest wall, lymphangitis, ulcerating tumor, number of recurrence episodes, year of treatment, total reRT dose, reRT field size, current chemotherapy, and current hormone treatment. The level of statistical significance was considered <0.05 for all analyses.

**Table 3:** Multivariable backward Wald stepwise binary logistic regression for ORR/Cox regression for LPFI

Covariate	ORR/*LPFI*	*P*-Value^b^	*P*-Value^a^	HR (95% CI)
ReBCEC >½ <¾ : ≥¾ chest wall	ORR	0.033	0.023	0.4 (0.2–1.0)
	*LPFI*	*NS*	*NS*	–
TI Primary tumor—current recurrence <med. : ≥med. (43 months)	ORR	0.019	0.020	2.7 (1.7–6.0)
	*LPFI*	*NS*	*NS*	–
Tumor ulceration prior to treatment Yes : no	ORR	0.003	0.001	3.3 (1.5–7.2)
	*LPFI*	*0.030*	*0.039*	*0.6 (0.4–1.0)*
Prior chemotherapy treatment Yes : no	ORR	NS	NS	–
	*LPFI*	*0.014*	*0.004*	*0.6 (0.3–0.9)*
Current chemotherapy treatment Yes : no	ORR	NS	0.017	*2.3 (1.2–4.8)*
	*LPFI*	NS	*0.018*	*0.6 (0.4–0.9)*

Upper values: ORR, *lower values: LPFI*

*ORR* overall response rate, *LPFI* local progression-free interval, *ReBCEC* current episode of recurrent breast cancer en cuirasse, *TI* time interval, *HR* hazard ratio, *CI* confidence interval, *NS* not significant, *med.* median

^a^Univariable

^b^Multivariable

## Results

### Treatment compliance

Overall, the reRT+HT treatment was well tolerated and 89% of patients finished the treatment according to plan. Eighteen out of 169 patients could not complete treatment: 14 due to distant progression, 3 because of toxicity, and 1 patient refused further treatment. Total reRT doses received by these patients varied from 4–36 Gy.

### Clinical outcome

**Fig. 1:**
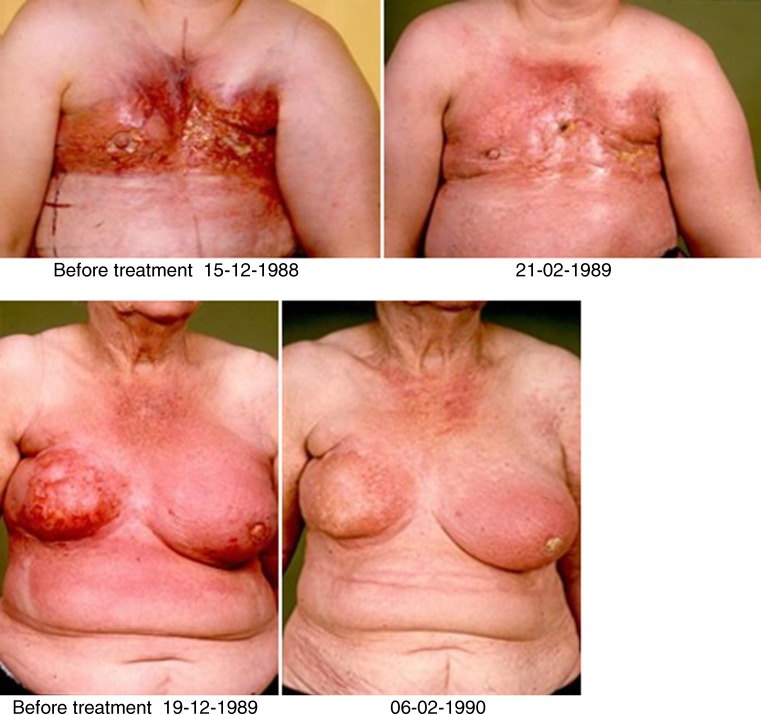
Example of 2 patients with clinical complete response (cCR) after reirradiation and hyperthermia (reRT+HT)

Overall clinical response rate (ORR) was 72% (30% complete responses and 42% partial responses). Fig. 1 shows two examples of patients with clinical complete response (cCR) after reRT+HT. In all, 22% had stable disease and 6% had progressive disease.

**Fig. 2:**
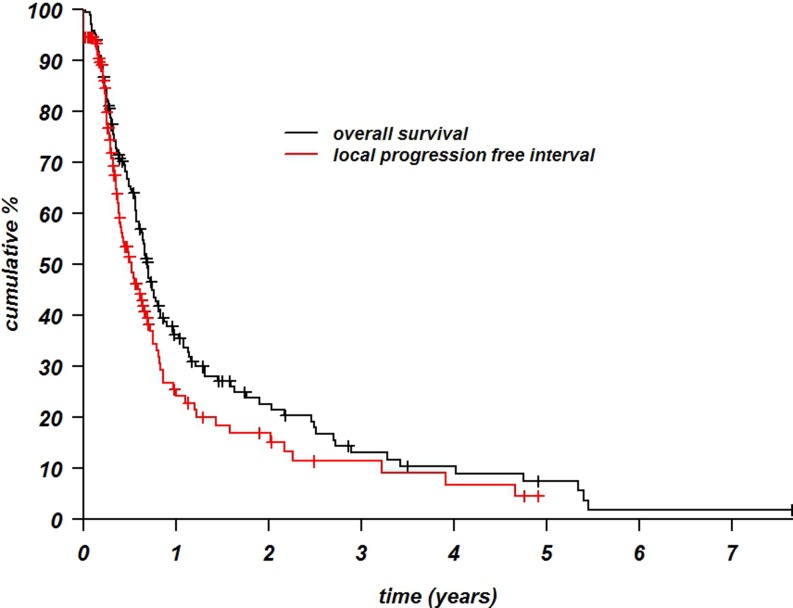
Local progression-free interval (LPFI) and overall survival rates according to Kaplan and Meier

The median overall FU time was 7 months (range 0.1–67 months). The 1‑year overall survival rate was 36% (95% CI 0.29051–0.452) with a median survival of 6.9 months (range 0.2–67.2 months). The 1‑year LPFI rate was 24% (95% CI 0.1674–0.349) with a median of 3.6 months (range 0–59 months; Fig. 2). Results from statistical analyses for ORR and LPFI are presented in Table 3. Only variables with significant values are shown. In multivariable analysis, a shorter time interval to recurrence, a large tumor area (≥¾ chest wall), and the presence of ulcerating tumor had a significant negative effect on ORR. The duration of LPFI was significantly decreased by the presence of ulcerating tumor and previous chemotherapy treatments in multivariable analysis. Both ORR and LPFI were thus significantly negatively affected by tumor ulceration (multivariable) and the addition of chemotherapy (univariable) to the current treatment episode (either before, during, or after the reRT+HT treatment).

### Toxicity

In 33% of patients, ≥grade 3 acute toxicity occurred, mostly moist desquamation and/or ulceration. One grade 4 acute ulceration occurred. The absolute ≥grade 3 late toxicity rate was 14%. The actuarial risk on ≥grade 3 late toxicity at 1 year was 18%. Late toxicity consisted mostly of ulceration. The number of acute and late grade 3 toxicities is reported in Table 4. One treatment related death due to pneumonitis was observed. None of the factors tested in the univariable and multivariable analysis was significantly related to overall ≥grade 3 late toxicity. Radiation related ulceration, the most dominant side effect in this population, was significantly related to the existence of tumor ulceration prior to treatment (*p* = 0.004, hazard ratio [HR] = 4.4).

**Table 4:** Grade 3 acute and late toxicity events (165 patients)

Toxicity^a^ acute/late	Grade 3
Dermatitis^b^	26/1
Ulceration	18/16
Pain	11/0
Blistering	2/2
Arm edema	1/2
Fibrosis	0/2
Telangiectasia	0/3
Brachial plexopathy	0/1
Pneumonitis	1/1

^a^Data missing for 4 patients

^b^Moist desquamation

## Discussion

We retrospectively evaluated clinical outcome and toxicity after reRT+HT in 169 patients treated for recurrent BCEC in two Dutch institutes. Our ORR of 72% is high considering refractory, extensive tumor growth. Forty-three percent of our patients with CR, PR, SD after treatment remained infield progression free until death or last follow-up.

Tumor size is a well-known prognostic factor for clinical outcome. Even in our population with very large tumor sizes, this is still an important factor for treatment response. Similar studies on reRT+HT for patients with smaller irresectable recurrent breast cancer, e. g., ≤½ ipsilateral chest wall, showed an ORR rate of 86% and a CR rate of 58% [28]. The meta-analysis on reRT+HT for locoregional recurrent breast cancer by Datta et al. resulted in a CR of 67% in 779 patients from 16 retrospective, single- or two-arm studies. These relatively high response rates resulted from the inclusion of studies on small, single lesions [19].

Other treatment options for patients with refractory inoperable recurrent breast cancer rarely report on locoregional tumor response. Two studies reported on locoregional tumor response after systemic treatment combinations for refractory inoperable recurrent breast cancer. ORR rates were 22 and 51% [9, 10]. Despite lower locoregional tumor load, these rates are lower than ours. The only other currently available treatments are of systemic nature and less effective in the palliative setting for irresectable locoregional recurrent breast cancer compared to reRT+HT. Response rates and treatments compliance were lower [9, 10] and side-effect- and treatment-related deaths higher, compared to our studies (Supplement 1 and 2, including references).

Our statistical analyses suggest that giving chemotherapy in the same treatment episode, either before, during, or after reRT+HT treatment adversely affects local palliation. Yet, 35% of our patients received chemotherapy for prior recurrences or for the current episode, in the absence of distant metastases. We think that reRT+HT should definitively be considered as part of standard palliative treatment regimens and should be part of the curative regimen for isolated locoregional recurrences as well.

Studies have shown HT not to enhance reRT toxicity [4, 29, 30]. Our current reRT+HT late ≥grade 3 toxicity rate (14%) is comparable to the rate published previously for smaller tumors, e. g., 18% [28]. There was, however, an increase in early ≥grade 3 toxicity from 24% for tumor areas ≤½ ipsilateral chest wall [28] to 33% for the larger tumors included in this study. Due to the lower number of patients and survival rate in this study, differences in late toxicity rates are difficult to detect. The increase in acute toxicity in our current study population might be related to the need for larger radiation volumes and the high frequency of tumor ulceration prior to treatment (52%). We did not find prognostic factors for overall toxicity in this patient population due to differences in patient characteristics and differences in effects of previous treatments. The heterogeneity in these cumulative effects determines susceptibility for subsequent treatment and is therefore not predictable, but remains related to individual patient characteristics.

There was, however, a significant relation between tumor ulceration before treatment and the development of radiation ulcera after reRT+HT, although it might be difficult to retrospectively determine cause or effect.

Another treatment regimen might be more beneficial for the group of 20 (12%) patients without treatment response or with local recurrence during follow-up, who developed a ≥grade 3 treatment-related ulceration. Small reRT fields and a low total reRT dose + HT aiming at reducing tumor burden without risk of severe side-effects should be considered for these patients, especially in view of the low survival rates. In case of subsequent recurrence, these patients could then be retreated using the same strategy increasing the palliative value of the treatment, as reported by Notter et al. [31]. A subgroup of patients who might benefit from this option are patients with ulcerating tumors who, according to our statistical analysis, have a significantly lower chance of treatment response, and are at higher risk for a subsequent infield recurrence as well as radiation-induced severe ulceration.

A focus shift might be needed to increase benefit for a larger number of patients with poor prognosis and low survival rates. Locoregional tumor growth can be extreme and often accompanied by ulceration. Focus in study design and in the clinical decision process is therefore on treatments that might sustainably reduce tumor load or prolong life. Less attention is paid to the risk of developing severe side effects after treatment and the effect hereof on quality of life (QoL). QoL assessments are frequently performed for clinical studies involving systemic treatments. Notably, QoL assessments have never been performed for reRT+HT studies on breast cancer and should be part of future clinical trials and incorporated in daily clinical practice.

## Conclusion

ReRT+HT provides sustainable palliative tumor control, despite refractory, extensive tumor growth. Compared to currently available systemic treatment options reRT+HT is more effective with less toxicity.

## Caption Electronic Supplementary Material

Literature review on side effects after systemic treatment for recurrent/metstatic breast cancer
