# Supplementary material for: Reirradiation + hyperthermia for recurrent breast cancer en cuirasse
Source: Strahlenther Onkol. 2017 Dec 20;194(3):206–14. doi: 10.1007/s00066-017-1241-7 (PMC5847022; doi:10.1007/s00066-017-1241-7)
Supplement: Supplementary file 1 — Literature review on side effects after systemic treatment for recurrent/metstatic breast cancer [file 66_2017_1241_MOESM1_ESM.doc]

**Supplement 1.**

**Reported common grade** 3+4 adverse events (CTCAE version 3 or 4) after systemic treatment for refractory inoperable locoregional recurrent/metastatic breast cancer.

| **Phase II-III trials** | CT | CT+CT | B | CT+B | LCT+CT | CT+CT+B | LCT+CT+B | Total |
| --- | --- | --- | --- | --- | --- | --- | --- | --- |
| Number of studies | 12 | 6 | 1 | 8 | 3 | 4 | 1 | 35 |
| Mean number of patients  (range) | 200  (30-503) | 205  (59-369) | 92 | 165  (41-284) | 153  (41-377) | 129  (50-255) | 46 | 141 |
| **AE** | **Mean in % (range)** | | | | | | | |
| Hematologic |  |  |  |  |  |  |  |  |
| Anemia | 6 (0-18) | 10 (0-22) | 0 | 2 (0-5) | 10 (2-16) | 11 (1-23) | 11 | 7^†^ |
| Neutropenia | 40 (6-66) | 60 (49-66) | 1 | 16 (39-2) | 37 (22-57) | 47 (20-67) | 35 | 34^†^ |
| Granulocytopenia | 0 | 10 (0-33) | 0 | 0 | 0 | 0 | u | 2^‡^ |
| Febrile neutropenia | 4 (0-11) | 25 (0-11) | 0 | 9 (0-16) | 7 | 1 (0-3) | u | 8^§^ |
| Leucopenia | 13 (0-49) | 19 (4-41) | 0 | 5 (0-8) | 17(7-25) | 11 (7-16) | 17 | 12^†^ |
| Thrombocytopenia | 4 (0-26) | 11 (0-27) | 0 | 1 (0-5) | 20 (0-34) | 18 (2-37) | 18 | 10^†^ |
| Gastrointestinal |  |  |  |  |  |  |  |  |
| Diarrhea | 3 (0-9) | 3 (0-6) | 0 | 5 (1-13) | 1 (0-2) | 2 (0-6) | 0 | 2^¦^ |
| Nausea | 2 (0-3) | 2 (0-4) | 0 | 2 (0-4) | <1 (0-1) | 3 (0-6) | 9 | 3^¦^ |
| Stomatitis / Mucositis | 2 (0-7) | 3 (0-6) | 3 | 4 (0-8) | 5 (0-12) | 1 (0-5) | 2 | 3^¶^ |
| Vomiting | 1 (0-3) | 2 (1-4) | 0 | 2 (0-6) | 2 (0-5) | 2 (0-3) | 4 | 2^¦^ |
| General disorders |  |  |  |  |  |  |  |  |
| Asthenia/fatigue | 8 (4-19) | 9 (2-19) | 1 | 7 (2-17) | 8 (2-14) | 6 (2-8) | 13 | 8 |
| Pyrexia | <1 (0-1) | <1 (0-1) | 0 | <1 (0-2) | 1 | <1 (0-1) | u | 1^‡^ |
| Pain | 3 (0-8) | 4 (0-9) | 0 | 3 (0-5) | 0 | 2 (0-4) | u | 2^‡^ |
| Peripheral edema | <1 (0-3) | <1 (0-2) | 0 | <1 (0-1) | u | <1 (0-1) | u | 1^**^ |
| Dyspnea | 1 (0-4) | 2 (0-4) | 0 | 1 (0-3) | 1 | 3 (0-7) | u | 1^‡^ |
| Arthralgia/Myalgia | 2 (0-11) | 2 (0-3) | 0 | 1 (0-4) | 0 | 1 (0-2) | u | 1^‡^ |
| Metabolism and Nutrition |  |  |  |  |  |  |  |  |
| Anorexia | 1 (0-3) | 1 (0-3) | 0 | <1 (0-2) | 1 (0-2) | 0 | 0 | 1^¶^ |
| Increased ALT | 0 | 2 (0-6) | 1 | <1 (0-2) | u | 3 (0-6) | u | 1^**^ |
| Increased AST | 0 | 2 (0-5) | 0 | 0 | u | 2 (0-6) | u | 1^#^ |
| Proteinuria | 0 | 0 | 4 | 2 (0-4) | 0 | 3 (0-10) | u | 2^§^ |
| Nervous system |  |  |  |  |  |  |  |  |
| Headache | < 1 (0-3) | <1 (0-2) | 0 | 1 (0-4) | 1 | 1 (0-3) | u | 1^‡^ |
| Dizziness | 0 | <1 (0-1) | 0 | 0 | 0 | <1 (0-1) | u | <1^‡^ |
| Peripheral neuropathy | 5 (0-15) | 4 (0-22) | 0 | 8 (1-27) | 1 | 0 | u | 3^‡^ |
| Skin and subcutaneous tissue |  |  |  |  |  |  |  |  |
| Alopecia | <1 (0-4) | 0 | 0 | 1 (0-4) | 0 | 0 | 0 | <1^¶^ |
| Hand-foot syndrome | 3 (0-17) | 5 (0-17) | 0 | 10 (0-31) | 8 (0-25) | 9 (0-34) | 0 | 5^¦^ |
| Nail disorder | <1 (0-4) | <1 (0-6) | 1 | 2 (0-7) | <1 | 0 | u | 1^‡^ |
| Cardiovascular |  |  |  |  |  |  |  |  |
| Embolism | <1 (0-3) | 0 | 0 | 3 (0-4) | 0 | 3 (0-10) | u | 1^‡^ |
| Hypertension | 0 (0-1) | 0 | 3 | 5 (0-13) | 0 | 4 (0-17) | u | 2^‡^ |
| CHF | 0 (0-1) | <1 (0-1) | 0 | <1 (0-1) | 1 | 0 | u | < ^††^ |
| Myocardial infarction | 0 | 0 | 0 | <1 (0-2) | 0 | 0 | u | <1^‡^ |

Only data for common AEs ( ≥ 2-20% population). AE = Adverse Event. CT = Chemotherapy. LCT = liposomal Chemotherapy. B = Biological agent. AE (any grade). U = unknown. ALT = Alanine Amino Transferase. AST = Aspartate aminotransferase. CHF = Congestive heart failure. ^†^ unknown for 2/35 studies. ^‡^ unknown for 6/35 studies. ^§^ unknown for 7/35 studies. ^¦^ unknown for 1/35 studies. ^¶^ unknown for 3/35 studies. ^#^ unknown for 9/35 studies. ^**^ unknown for 8/35 studies. ^††^ unknown for 5/35 studies.

Single chemotherapy regimens: Ixabepilone (3 studies), Eribulin (4 studies), Docetaxel (2 studies), Capecitabine (2 studies) and Satraplatin. Combination chemotherapy regimens: Ixabepilone+Capecitabine, Docetaxel+Capecitabine (2 studies), Gemcitabine+Carboplatin (3 studies). Biological agents: Bevacizumab. Biological agents added to mono chemotherapy: Bevacizumab+Capecitabine, Eribulin+Trastuzumab, Lapatinib+Capecitabine, Bevacizumab+Docetaxel (2 studies), Paclitaxel+Bevacizumab, Paclitaxel+Trastuzumab, Docetaxel+Trastuzumab. Mono chemotherapy combined with liposomal drugs: liposomal Doxorubicin (PLD)+Docetaxel, PLD+Carboplatin (2studies). Biological agents added to combination chemotherapy: Gemcitabine+Carboplatin+Trastuzumab, Gemcitabine+Carboplatin+Iniparib (2 studies), Paclitaxel+Capecitabine+Bevacizumab. Combined (liposomal) chemotherapy plus biological agent: PLD+Carboplatin+Trastuzumab. [1-19]

**Supplement 2.**

**Summary of supplement 1**

| **Phase II-III trials** | | CT | | CT+CT | | B | | CT+B | | LCT+CT | | CT+CT+B | | LCT+CT+B | | Total |
| --- | --- | --- | --- | --- | --- | --- | --- | --- | --- | --- | --- | --- | --- | --- | --- | --- |
| Number of studies | | 12 | | 6 | | 1 | | 8 | | 3 | | 4 | | 1 | | 35 |
| Mean number of patients  (range) | | 200  (30-503) | | 205  (59-369) | | 92 | | 165  (41-284) | | 153  (41-377) | | 129  (50-255) | | 46 | | 141 |
| Overall AE grade 3/4 | | 59 (28-72) | | 74 (59-86) | | 27 | | 62 (46-78) | | 78 | | 88 (86-89) | | u | | 65^**^ |
| Death^*^ | 1 (0-4) | | 8 (0-33) | | 1 | | 2 (1-2) | | 0 | | 1 (0-2) | | 0 | | 2^¦^ | |
| Discontinuation^*^ | | 11 (0-33) | | 21 (12-31) | | 13 | | 15 (9-21) | | 15 (12-20) | | 19 (10-35) | | 15 | | 16^¦^ |
| Dose adjustment^s*^ | | 35 (12-63) | | 43 (11-78) | | 5 | | 17 (1-35) | | 31 | | 45 (19-84) | | u | | 29^#^ |

Only data for common AEs ( ≥ 2-20% population). AE = Adverse Event. CT = Chemotherapy. LCT = liposomal Chemotherapy. B = Biological agent. U = unknown. Dose adjustments = reduction, omission or modification. ^*^Due to AEs. ^¦^ unknown for 1/35 studies. ^#^ unknown for 9/35 studies. ^**^ unknown for 17/35 studies [1-19]

**References**

1. Perez EA, Lerzo G, Pivot X, Thomas E, Vahdat L, Bosserman L, Viens P, Cai C, Mullaney B, Peck R, Hortobagyi GN (2007) Efficacy and safety of ixabepilone (BMS-247550) in a phase II study of patients with advanced breast cancer resistant to an anthracycline, a taxane, and capecitabine. J Clin Oncol 25 (23):3407-3414.

2. Smith JW, 2nd, Vukelja S, Rabe A, Wentworth-Hartung N, Koutrelakos N, Shao SH, Whittaker T, Wang Y, Asmar L, McDowell DO, Mukhopadhyay P, O'Shaughnessy J (2013) Phase II randomized trial of weekly and every-3-week ixabepilone in metastatic breast cancer patients. Breast Cancer Res Treat 142 (2):381-388.

3. Vahdat LT, Pruitt B, Fabian CJ, Rivera RR, Smith DA, Tan-Chiu E, Wright J, Tan AR, Dacosta NA, Chuang E, Smith J, O'Shaughnessy J, Shuster DE, Meneses NL, Chandrawansa K, Fang F, Cole PE, Ashworth S, Blum JL (2009) Phase II study of eribulin mesylate, a halichondrin B analog, in patients with metastatic breast cancer previously treated with an anthracycline and a taxane. J Clin Oncol 27 (18):2954-2961.

4. Cortes J, Vahdat L, Blum JL, Twelves C, Campone M, Roche H, Bachelot T, Awada A, Paridaens R, Goncalves A, Shuster DE, Wanders J, Fang F, Gurnani R, Richmond E, Cole PE, Ashworth S, Allison MA (2010) Phase II study of the halichondrin B analog eribulin mesylate in patients with locally advanced or metastatic breast cancer previously treated with an anthracycline, a taxane, and capecitabine. J Clin Oncol 28 (25):3922-3928.

5. Cortes J, O'Shaughnessy J, Loesch D, Blum JL, Vahdat LT, Petrakova K, Chollet P, Manikas A, Dieras V, Delozier T, Vladimirov V, Cardoso F, Koh H, Bougnoux P, Dutcus CE, Seegobin S, Mir D, Meneses N, Wanders J, Twelves C, investigators E (2011) Eribulin monotherapy versus treatment of physician's choice in patients with metastatic breast cancer (EMBRACE): a phase 3 open-label randomised study. Lancet 377 (9769):914-923.

6. Sparano JA, Makhson AN, Semiglazov VF, Tjulandin SA, Balashova OI, Bondarenko IN, Bogdanova NV, Manikhas GM, Oliynychenko GP, Chatikhine VA, Zhuang SH, Xiu L, Yuan Z, Rackoff WR (2009) Pegylated liposomal doxorubicin plus docetaxel significantly improves time to progression without additive cardiotoxicity compared with docetaxel monotherapy in patients with advanced breast cancer previously treated with neoadjuvant-adjuvant anthracycline therapy: results from a randomized phase III study. J Clin Oncol 27 (27):4522-4529.

7. Miles DW, Chan A, Dirix LY, Cortes J, Pivot X, Tomczak P, Delozier T, Sohn JH, Provencher L, Puglisi F, Harbeck N, Steger GG, Schneeweiss A, Wardley AM, Chlistalla A, Romieu G (2010) Phase III study of bevacizumab plus docetaxel compared with placebo plus docetaxel for the first-line treatment of human epidermal growth factor receptor 2-negative metastatic breast cancer. J Clin Oncol 28 (20):3239-3247.

8. Hortobagyi GN, Gomez HL, Li RK, Chung HC, Fein LE, Chan VF, Jassem J, Lerzo GL, Pivot XB, Hurtado de Mendoza F, Xu B, Vahdat LT, Peck RA, Mukhopadhyay P, Roche HH (2010) Analysis of overall survival from a phase III study of ixabepilone plus capecitabine versus capecitabine in patients with MBC resistant to anthracyclines and taxanes. Breast Cancer Res Treat 122 (2):409-418.

9. Cameron D, Casey M, Press M, Lindquist D, Pienkowski T, Romieu CG, Chan S, Jagiello-Gruszfeld A, Kaufman B, Crown J, Chan A, Campone M, Viens P, Davidson N, Gorbounova V, Raats JI, Skarlos D, Newstat B, Roychowdhury D, Paoletti P, Oliva C, Rubin S, Stein S, Geyer CE (2008) A phase III randomized comparison of lapatinib plus capecitabine versus capecitabine alone in women with advanced breast cancer that has progressed on trastuzumab: updated efficacy and biomarker analyses. Breast Cancer Res Treat 112 (3):533-543.

10. Smith JW, 2nd, McIntyre KJ, Acevedo PV, Encarnacion CA, Tedesco KL, Wang Y, Asmar L, O'Shaughnessy JA (2009) Results of a phase II open-label, nonrandomized trial of oral satraplatin in patients with metastatic breast cancer. Breast Cancer Res Treat 118 (2):361-367.

11. Buzdar AU, Xu B, Digumarti R, Goedhals L, Hu X, Semiglazov V, Cheporov S, Gotovkin E, Hoersch S, Rittweger K, Miles DW, O'Shaughnessy J, Tjulandin S, group NOt (2012) Randomized phase II non-inferiority study (NO16853) of two different doses of capecitabine in combination with docetaxel for locally advanced/metastatic breast cancer. Ann Oncol 23 (3):589-597.

12. O'Shaughnessy J, Schwartzberg L, Danso MA, Miller KD, Rugo HS, Neubauer M, Robert N, Hellerstedt B, Saleh M, Richards P, Specht JM, Yardley DA, Carlson RW, Finn RS, Charpentier E, Garcia-Ribas I, Winer EP (2014) Phase III study of iniparib plus gemcitabine and carboplatin versus gemcitabine and carboplatin in patients with metastatic triple-negative breast cancer. J Clin Oncol 32 (34):3840-3847.

13. O'Shaughnessy J, Osborne C, Pippen JE, Yoffe M, Patt D, Rocha C, Koo IC, Sherman BM, Bradley C (2011) Iniparib plus chemotherapy in metastatic triple-negative breast cancer. N Engl J Med 364 (3):205-214.

14. Loesch D, Asmar L, McIntyre K, Doane L, Monticelli M, Paul D, Vukelja S, Orlando M, Vaughn LG, Zhan F, Boehm KA, O'Shaughnessy JA (2008) Phase II trial of gemcitabine/carboplatin (plus trastuzumab in HER2-positive disease) in patients with metastatic breast cancer. Clin Breast Cancer 8 (2):178-186.

15. Gligorov J, Doval D, Bines J, Alba E, Cortes P, Pierga JY, Gupta V, Costa R, Srock S, de Ducla S, Freudensprung U, Mustacchi G (2014) Maintenance capecitabine and bevacizumab versus bevacizumab alone after initial first-line bevacizumab and docetaxel for patients with HER2-negative metastatic breast cancer (IMELDA): a randomised, open-label, phase 3 trial. Lancet Oncol 15 (12):1351-1360.

16. Wilks S, Puhalla S, O'Shaughnessy J, Schwartzberg L, Berrak E, Song J, Cox D, Vahdat L (2014) Phase 2, multicenter, single-arm study of eribulin mesylate with trastuzumab as first-line therapy for locally recurrent or metastatic HER2-positive breast cancer. Clin Breast Cancer 14 (6):405-412.

17. Lam SW, de Groot SM, Honkoop AH, Jager A, ten Tije AJ, Bos MM, Linn SC, van den Bosch J, Kroep JR, Braun JJ, van Tinteren H, Boven E, Dutch Breast Cancer Research G (2014) Paclitaxel and bevacizumab with or without capecitabine as first-line treatment for HER2-negative locally recurrent or metastatic breast cancer: a multicentre, open-label, randomised phase 2 trial. Eur J Cancer 50 (18):3077-3088.

18. Lang I, Bell R, Feng FY, Lopez RI, Jassem J, Semiglazov V, Al-Sakaff N, Heinzmann D, Chang J (2014) Trastuzumab retreatment after relapse on adjuvant trastuzumab therapy for human epidermal growth factor receptor 2-positive breast cancer: final results of the Retreatment after HErceptin Adjuvant trial. Clin Oncol (R Coll Radiol) 26 (2):81-89.

19. Collea RP, Kruter FW, Cantrell JE, George TK, Kruger S, Favret AM, Lindquist DL, Melnyk AM, Pluenneke RE, Shao SH, Crockett MW, Asmar L, O'Shaughnessy J (2012) Pegylated liposomal doxorubicin plus carboplatin in patients with metastatic breast cancer: a phase II study. Ann Oncol 23 (10):2599-2605.
